# GPs’ use of video and telephone consultation: implications for physical activity promotion for older adults. A mixed-methods study

**DOI:** 10.3399/BJGPO.2023.0256

**Published:** 2025-08-13

**Authors:** Christopher Thomas Callaghan, Conor Cunningham, Roger O’Sullivan

**Affiliations:** 1 Institute of Public Health, Belfast, Northern Ireland, UK; 2 School of Health Science, Ulster University, Belfast, Northern Ireland, UK; 3 The Bamford Centre for Mental Health and Wellbeing, Ulster University, Belfast, Northern Ireland, UK

**Keywords:** general practitioners, video, telephone, remote consultation

## Abstract

The COVID-19 pandemic led GPs to adopt video consultation and telephone consultation (VC/TC) as part of routine practice. The potential impact on physical activity (PA) promotion and prescription for older adults, in routine practice, was unknown.

**Aim:**

To understand how VC/TC have impacted on the routine promotion of PA to older adults in GP consultations.

**Design & setting:**

A mixed-methods, cross-sectional study of GPs was conducted in the Republic of Ireland (RoI) and Northern Ireland (NI) in 2020/2021.

**Method:**

An online survey and interviews were conducted with GPs that explored awareness of PA guidelines, PA promotion during consultations to older adults (aged ≥65 years), and the impact on routine practice of moving to VC/TC during the pandemic.

**Results:**

GPs from across the island of Ireland (NI and RoI) agreed that PA promotion is part of routine practice. Analysis of interviews with GPs highlighted a need to develop practitioners’ knowledge of the guidelines, and themes emerged around the use of VC/TC in routine practice. The positive themes highlighted that VC/TC enabled GPs to consult with a greater number of patients compared with face-to-face consultation (FTFC). Barriers to using VC/TC included decreased visual assessments of patients face to face (FTF).

**Conclusion:**

GPs are continuing to use VC/TC at the initial consultation stage, but the broader and longer-term implications on PA promotion with older adults are unknown.

## How this fits in

Video and telephone consultation (VC/TC) was adopted widely in the UK and the RoI by GPs in response to the COVID-19 pandemic. Remote consultations have continued to be used by default by many GPs following the pandemic. However, few studies have examined the implications related to the increased use and challenges of VC/TC as a tool for GP consultations. Given the projections for a shortage in general practice provision, it is anticipated that VC/TC will continue to be used in the initial assessment of patients. Therefore, it is imperative for research studies to explore the impact of VC/TCs on patient care.

## Introduction

In 2020, VC/TCs were widely rolled out across general practice owing to public health and social measures (PHSM) introduced during the COVID-19 pandemic.^1^ Although we are now post-pandemic, many GPs have maintained non-face-to-face (FTF) patient assessments.^2,3^ Before the pandemic, VC/TCs were previously used across a range of other areas of health care, including mental health and musculoskeletal pain management services. These consultations were found to be effective in treating mood and eating disorders, resulting in a reduction of symptoms, and were also considered as effective as in-person consultations for musculoskeletal pain management support.^4,5^ Research in primary care in the UK found that VC/TC consultations are similar in length, content, and quality to face-to-face consultations (FTFCs). However, both appear less ‘information rich’ than FTFCs, and are suited to health issues or conditions not requiring a physical examination. In addition, VC/TCs are more likely to be utilised by younger patients.^6^

The implications of this move in relation to physical activity (PA) promotion in routine practice and in health services, however, is not fully understood. International guidelines recommended, at the time of this research, that older adults (aged ≥65 years) should aim to do at least 150 minutes of moderate intensity or 75 minutes of vigorous intensity aerobic activity throughout the week, with muscle strengthening and multicomponent balance training on 2 or more days per week.^7^ Research shows that physically active older adults experience healthier ageing trajectories,^8^ resulting in a better quality of life and a reduced risk of all-cause and cardiovascular mortality, breast and prostate cancer, fractures, recurrent falls, disability, cognitive decline, dementia, and depression.^8,9^

Sedentary lifestyle behaviour and physical inactivity are the leading modifiable risk factors for cardiovascular disease and all-cause mortality.^10^ Consequently, this stage of life (aged ≥65 years) is a particularly important period for encouraging PA to improve quality of life and slow progression of disease and disability.^11^ Furthermore, regular moderate-to-vigorous PA has been associated with lower rates of depression.^9,12^ For many, ageing is defined by a rapid decline in levels of PA; loss of mobility; loss of functional independence; increased frailty; and premature mortality.^13^ Consequently, as we are living longer, this is an important period to promote PA, which aids mobility and daily function and helps slow the progression of disability and disease.^13,14^

GPs are in an ideal position to promote PA interventions, serving as a valuable source of information and guidance.^15^ Evidence indicates that GPs can have a positive role in promoting behaviour change by assessing patients’ levels of PA on a regular basis, discussing benefits of PA and also signposting to local programmes.^16–18^^,^ However, recent research with GPs has identified a need for continuing professional education and skill development in routine clinical practice along with the provision of additional in-service training and evidence-informed resources to effectively support the promotion of PA in routine practice to the older adult population.^18^ Barriers identified in research to promoting PA have been reported by GPs as including lack of awareness, expertise, time, and incentive.^15,18–20^

To the best of our knowledge, our study is unique and it examines cross-sectional data from GPs across NI and the RoI during 2020/2021. The aim of the present study is to understand how VC/TC have impacted on the routine promotion of PA to older adults in general practice consultations.

## Method

This article is based on a mixed-methods, cross-sectional study of healthcare professionals (GPs, physiotherapists, occupational therapists, and nurses) in the RoI and NI. The study design and sampling methodology were previously set out in detail by Cunningham and O’Sullivan.^15,18^ The focus of this article is on GPs and uses contextual and illustrative GP data from Cunningham and O’Sullivan ^15,18^ as well as previously unpublished data.

### Study design

GPs were recruited with support from the Royal College of General Practitioners in NI, the Irish College of General Practitioners in the RoI, and other key organisations. A research advisory group was established to help develop, refine and pilot the survey and interview questions. The survey ran from mid-August to mid-October 2020 and the semi-structured interviews took place between November 2020–March 2021.^15,18^

### Data collection

Participants were invited to complete an online survey. It consisted of: **Section one**, which covered demographic and employment data, as well as GPs’ self-reported knowledge on levels of PA guidelines and awareness of resources to facilitate knowledge and practice development; and **Section two,** which was developed using the Theoretical Domains Framework (TDF).^21,22^ The TDF is an integrative framework of theories of behaviour change developed to identify influences on behaviour in the implementation of evidence-based recommendations. This section of the survey covered the GP’s behaviour in assessment, discussion, and prescription of PA in routine practice as well as the implications of the COVID-19 pandemic on older adults’ levels of PA, and their views on the role of PA promotion to older adults.

The qualitative component used semi-structured, online interviews. Participants were recruited through professional bodies and networks and those who responded to the survey were also invited to participate. To ensure that each profession was represented, a purposeful sampling method was used. Interviews were guided by the TDF and covered themes including: knowledge of PA guidelines; factors impacting assessment and prescription of PA; opportunities to promote PA in day-to-day practice; and barriers and facilitators to PA promotion. A total of *n* = 10 GPs participated (Table 1).

**Table 1.** Characteristics of the interviewees

Healthcare profession	Practice experience	Self-rated knowledge of guidelines for PA (1–10)	Current practice setting
NIGP1	GP: 0+ years’ general practice experience	^a^No Response	Community practice
NIGP2	GP: 20+ years’ general practice experience	^a^No response	Community practice
NIGP3	GP: 20+ years’ general practice experience	5	Community practice
NIGP4	GP: 5+ years’ experience	4	Community practice
NIGP5	GP in training (clinical and academic training programme)	10	Community practice
RoIGP1	GP: 10+ years’ practice experience	7/8	Community practice
RoIGP2	GP: 10+ years’ general practice experience	8	^a^Unknown
RoIGP3	GP: 10+ years’ general practice experience	2	Community practice
RoIGP4	GP: no data obtained	6/7	Community practice
RoIGP5	GP: no data obtained	7/8	^a^Unknown

^a^Unknown, no response.

NI = Northern Ireland. RoI = Republic of Ireland.

The study was conducted according to the guidelines of the Declaration of Helsinki and approved by an Independent Peer Review panel.

### Analysis

A descriptive analysis was conducted on all eligible returned surveys in the overall study.^15^ An analysis of the data was performed using SPSS (version 24). Pearson’s χ^2^ tests were used to compare knowledge of guidelines for PA with assessment, discussion, prescription, and signposting of PA in routine practice. Statistical significance was set at *P* value <0.05. Qualitative data, in the overall study, were analysed using NVivo software (version 12.0). The data were mapped onto relevant TDF domains.^22^ The mapping used a deductive thematic analysis approach. All differences were discussed, and a consensus was reached to ensure the coding and mapping were appropriate.^18^ To guide the reporting of the qualitative data, the STROBE Checklist was used.^23^

In this article, descriptive statistics using percentages are presented on GPs to profile participants and their responses to the survey. In relation to the qualitative analysis for this article, interviewees had been asked a question on the theme of how COVID-19 PHSM impacted practice and PA promotion and this was included in the analysis; a common theme was the discussion of the move to VC/TCs. Inductive thematic analysis was used by RO and CTC to review the GP interviews, and the responses were categorised into either positive experiences or challenges. Again, any differences were discussed, and a consensus reached.

## Results

### Participant characteristics

A total of 36 GPs responded to the survey and were included in the analysis. The participant characteristics are presented in Table 2. Of the responders, 14 (38.9%) were male and 20 (55.6%) were female, two (5.6%) preferred not to say. A total of 38.9% of GPs (*n* = 14) reported that they had ≥26 years of practice experience. Most responders worked in the public sector (91.7%, *n* = 33). Just over half of GP responders (51.4%, *n* = 18) achieved the recommended level of moderate intensity PA over a week.

**Table 2.** Characteristics of GP survey responders

Characteristic	Frequency, *n* (%)
Total responders	36
**Sex**	
Male	14 (38.9)
Female	20 (55.6)
Prefer not to say	2 (5.6)
**Years qualified**	
0–5	N/A
6–10	4 (11.1)
11–15	5 (13.9)
16–20	6 (16.7)
21–25	7 (19.4)
≥26	14 (38.9)
**Healthcare setting**	
Primary	27 (75.0)
Secondary	7 (19.4)
Other	2 (5.6)
**Health sector**	
Public	33 (91.7)
Private	3 (8.4)
Other	N/A
**Region**	
Northern Ireland	14 (38.9)
Republic of Ireland	22 (61.1)
Total	36
**Physical activity levels**	
Active	18 (51.4)
Inactive	17 (48.6)
Total	*35

* 1 GP didn’t respond. Please note due to rounding, totals may not always add up to 100%.

### GPs’ awareness of physical activity guidelines and resources

GPs were asked in this survey about their level of awareness of the content and objectives of national PA guidelines in their jurisdiction, 22.2% agreed (*n* = 8) and 33.3% somewhat agreed (*n* = 12) that they were aware (Table 3). GPs were also asked about their knowledge of three specific components of PA recommendations for older adults (number of minutes per week of moderate intensity PA, vigorous intensity PA, and the number of days of strength, balance, and flexibility activities recommended for optimal health benefits). Of the GPs participating in this survey, 41.7% (*n* = 15) reported knowing how many weekly minutes of moderate intensity PA were recommended for older adults, 19.4% (*n =* 7) indicated they were aware of the number of minutes recommended of weekly vigorous intensity PA for older adults. In addition 27.8% (*n* = 10) of GP responders indicated they knew the frequency of strength, balance, and flexibility training recommended for older adults. Overall correct answers to these three questions were lower and a minority 5.6% (*n =* 2) identified all three components of the PA guidelines for older adults correctly. Just over 11.1% (*n* = 4) of GPs had awareness of resources to facilitate their knowledge development and prescription of PA with patients as a part of routine care. Resources cited included government health department websites, professional body websites, health-profession-specific websites.

**Table 3.** GPs’ awareness of physical activity guidelines and resources: knowledge, understanding, and use of physical activity guidelines

Survey question	Answer	Frequency, ***n*** (**%**)
I am aware of the content and objectives of national guidelines for physical activity in my jurisdiction	Agree Somewhat agree Neither agree nor disagree Somewhat disagree Disagree Not stated	8 (22.2) 12 (33.3) 3 (8.3) 1 (2.8) 6 (16.7) 6 (16.7)
I am aware of the content and objectives of national guidelines for physical activity for older adults in my jurisdiction	Agree Somewhat agree Neither agree nor disagree Somewhat disagree Disagree Not stated	8 (22.2) 7 (19.4) 4 (11.1) 4 (11.1) 7 (19.4) 6 (16.7)
**Awareness of specific component(s) of physical activity for older adults**		
Do you know how many minutes of moderate intensity physical activity that the national guidelines recommend per week for older adults in your jurisdiction?	Yes Correctly answered No Not stated	15 (41.7) 9 (25.0) 15 (41.7) 6 (16.7)
		
Do you know how many minutes of vigorous intensity physical activity that the national guidelines recommend per week for older adults in your jurisdiction?	Yes Correctly answered No Not stated	7 (19.4) 2 (5.6) 23 (63.9) 6 (16.7)
Do you know how many days per week that national guidelines in your jurisdiction recommend older adults perform strength, balance, and flexibility training?	Yes Correctly answered No Not stated	10 (27.8) 9 (25.0) 20 (55.6) 6 (16.7)
Correctly answered all three questions		2 (5.6)
**Awareness of resources**		
I am aware of resources (that is, online resources and toolkits) to facilitate my knowledge development and practice of discussions, assessment, and prescription of physical activity with patients as a part of routine care	Yes No Not stated	4 (11.1) 26 (72.2) 6 (16.7)

GPs were asked if PA guidelines had a place in routine practice consultation and if GPs have the time to implement PA consultations in routine practice. The responses are presented in Table 4. Half, 50% (*n* = 18) agreed PA guidelines have a place in routine practice consultation and 25% (*n* = 9) somewhat agree, while of the others who answered, 2.8% (*n* = 1) disagreed. Only 5.6% (*n* = 2) agreed and 8.3% (*n* = 3) somewhat agreed that GPs have the time to implement PA consultations in routine practice. However, 16.7% (*n* = 6) neither agreed nor disagreed, while 22.2% (*n* = 8) somewhat disagreed, and 30.6% (*n* = 11) stated they disagreed.

**Table 4.** GP views on PA guidelines’ place in routine practice

Survey question	Answer	Frequency, *n* (%)
Physical activity guidelines have a place in routine practice	Agree	18 (50)
	Somewhat agree	9 (25)
	Neither agree nor disagree	2 (5.6)
	Somewhat disagree	0 (0)
	Disagree	1 (2.8)
	Not stated	6 (16.7)
There is sufficient time allocated in day-to-day work (routine practice) to implement physical activity guidelines for adults/older adults	Agree	2 (5.6)
	Somewhat agree	3 (8.3)
	Neither agree nor disagree	6 (16.7)
	Somewhat disagree	8 (22.2)
	Disagree	11 (30.60)
	Not stated	6 (16.7)

Table 5 sets out the GPs’ views on the potential impact of PHSM on older adults’ levels of PA. Over half of GP responders (55.6%, *n* = 20), felt that PHSM introduced to prevent the spread of COVID-19, decreased the levels of PA in older adults. To the question “In the light of the COVID-19 pandemic — do you think that health professionals can play an increased role in promoting physical activity to older adults?”66.7% (*n* = 24) agreed and 33.3% (*n* = 12) were more likely to discuss physical activity with older adults as part of routine practice.

**Table 5.** The COVID-19 pandemic: implications for older adults’ physical activity

Survey question	Answer	Frequency, *n* (%)
Public health and social measures introduced to prevent the spread of COVID -19 have reduced older adults’ levels of physical activity	Agree	20 (55.6)
	Somewhat agree	5 (13.9)
	Neither agree nor disagree	2 (5.6)
	Somewhat disagree	3 (8.3)
	Disagree	0 (0)
	Not stated	6 (16.7)
In the light of the COVID-19 pandemic — do you think that health professionals can play an increased role in promoting physical activity to older adults?	Yes	24 (66.7)
	No	2 (5.6)
	Don’t know	3 (8.3)
	Not stated	7 (19.4)
Considering the COVID-19 pandemic—how likely are you to discuss physical activity with older adults as part of routine practice?	More likely	12 (33.3)
	Same as usual	15 (41.7)
	Less likely	3 (8.3)
	Not stated	6 (16.7)

### Interview results

A total of 10 GPs practicing in NI and the ROI were interviewed and included in the analysis. The participant characteristics are presented in Table 1. The responders included both male and female GPs practicing in NI and the ROI, in both urban and rural settings (at least half had 10 or more years of practice experience).

GPs’ illustrative quotations are presented in Table 6. To enhance our understanding of practice in the use of VC/TCs, we explored the opportunities and barriers in relation to the delivery of PA support to older adults in general practice consultations. Overall, GPs highlighted that the COVID-19 pandemic accelerated the utilisation of VC/TCs in routine practice; however, appropriate guidelines for integration were still to be rolled out at the time of the interviews.

**Table 6.** GP emergent themes on the application and integration of physical activity promotion in routine practice: pre and during the public health and social measures. Illustrative quotations

**Benefits of physical activity: illustrative quotations**
*‘You know that when we are prescribing physical activity, just because it isn’t coming in a capsule, doesn’t mean it probably has infinitely more benefits than the vast majority of treatments.’* (RoIGP3)
**Barriers to recommending physical activity in routine practice: illustrative quotations**
***‘*** *I’d certainly say that the message we’re best at promoting is around smoking cessation. So, we’ve got this pretty solid evidence that GPs are willing to give brief interventions on smoking cessation and that it works. Then following that alcohol is probably number two. I think physical activity is well down the list.’* (RoIGP2) *‘I’m very comfortable to talk to patients about* [physical activity]*, but I would be less comfortable to quote the guidance to them.’* (NIGP1)
**Initial training and continuing professional development on physical activity: illustrative quotation**
*‘Yeah, unfortunately there wasn’t actually a whole lot* [on physical activity] *to be honest with you. I distinctly remember doing a few lectures or professional competency sessions on health and exercise. Were kind of on really detailed calorie counting. Complex equations that I’ve never used since, unfortunately. Very minimal practical applications.’* (RoI GP4)
**Time barriers to recommending physical activity: illustrative quotations**
*‘No, because within the context of general practice, you don’t have that much time to you know, use tools and things, you know, there are lots of physical activity questionnaires and things, but they can be quite time -consuming and formulaic.’* (NIGP5)
**Benefits of video and telephone consultations: illustrative quotations**
*‘… Some* [GP practices] *… may go back to traditional surgeries — we won’t because we felt that we just couldn’t keep up with demand, and waiting times were long. We just feel this way is much more efficient …’* (NIGP2) *‘... there’s been a big change in consulting practice, in my practice and in GPs across the board, to a move to more remote consulting. So, that’s like telephone consulting primarily ... I personally feel that you have more time to give to the specific patient* [in a telephone consultation]*.’* (NIGP5)
**Barriers to video and telephone consultations in routine practice: illustrative quotations**
*‘I didn’t try and turn into a phone. I just, I stayed here and stayed open so that … people *[could]* come in and see me.’* (RoIGP01) *‘Sometimes you get a better read and a better rapport with the patient face-to-face and you know, that maybe is lost a little bit on telephone calls.’* (NIGP4)
**Barriers to recommendation physical activity in video and telephone consultations: illustrative quotations**
*‘So, doing remote consultations to me, I am missing some of that soft touch that we get when patients are in the room. So, it won’t be evident to me if I am on the phone and I can’t see if somebody is* [...] *overweight … So I am missing that, the activity bit, I can’t softly see if somebody is getting out of breath by walking into the room. There are all sorts of soft messages I can pick up on.’* (NIGP3)
**Assessing physical activity in routine practice: illustrative quotations**
*‘It is kind of just conversation. I would consider it a blind spot really for me. It would kind of be maybe a known unknown … I know that there must be assessment tools out there, but I just have never really incorporated them.’* (RoIGP4) *‘I suppose it depends on the flow of any particular consult as to how it might weave its way in there …. there is generally an assessment directly and indirectly as to where they are at in their current levels of exercise and then a sense of their understanding of it … ‘* (RoIGP3)
**Assessing physical activity in routine practice via video and telephone consultations: illustrative quotations**
*‘I suspect probably, in the phone conversations, exercise would probably come in less. I am not sure if I tested that theory, but my own feeling is that it is probably getting asked a bit less by me anyway.’* (RoIGP5) *‘I would think, certainly* [physical activity] *would come into all consultations, whether they’re telephone or face to face.’* (NIGP2)
**GPs’ professional role and identity: illustrative quotations**
*‘... I think they look to the GP as a source of expert advice on their health in general and I think we’re privileged that a lot of patients would kind of trust our guidance and our opinion. And so, yeah, I think that a GP is ideally placed for this’.* (RoIGP4) *‘I think we’ve grown up with this sort of “check with your doctor first before you undertake this activity” … That has been kind of a message that has been tagged on everything for years, so there has been an inherent fear …’* (NIGP1)

Opportunities from the use of VC/TCs include allowing more time for GPs to consult with a higher number of patients per day compared with FTFCs. Barriers for the use of VC/TCs include GPs not being able to carry out a close visual assessment, limiting conversations around PA assessment and of the patient’s level of fitness, thus restricting the opportunity to recommend PA interventions.

## Discussion

### Summary

In this article, we have highlighted GPs’ own insights and experiences on the increasing use of VC/TCs with patients in general practice. However, we need to develop our understanding of how VC/TCs may impact the promotion of PA for older adults in general practice consultations. Few studies have examined the implications related to the increased use and challenges of VC/TCs as a tool for GP consultations. Most prior studies on VC/TCs tend to have focused on secondary care settings, and overall studies focusing on VC/TCs in general practice have been limited.^24,25^ It is recognised that GPs can play an important role in advising patients about the benefits of leading a healthy and active lifestyle and the implications of being inactive.^18,26^ It is also recognised that increasing workloads in general practice require additional resources and staffing as well as new models of delivering community-based services to patients.^27^ This research found that GPs agree discussing PA is part of their job role. However, evidence from the responders highlights that suitable training had not been delivered to initiate conversations and discussions around PA with patients within a VC/TC context.

Drawing on the data, the results identified a range of common themes, including that VC/TCs allow GPs to consult with more patients over a daily period, and in some practices allow more time for screened patients who require a more detailed consultation. On the other side, a reduction of FTFCs raised a concern that rapport could be diminishing. While it is recognised if an older patient has a smartphone or suitable device, they are able to participate in VC/TCs, however there may be lost opportunities for vulnerable and marginalised groups who do not have access to smartphones or appropriate information and communication technology (ICT) equipment to access VC/TCs.^28^

### Strengths and limitations

This study gathered views from GPs during the COVID-19 pandemic and the transition to VC/TC. It included interviews and a survey in two jurisdictions and two different healthcare systems. The generalisability of the study findings should be considered, given the size of the study. For example selection bias may be present, as it is possible that GPs who are interested in and utilise PA promotion in routine practice may be more inclined to participate. Nonetheless, the overall patterns in this study align with other studies in this area.

### Comparison with existing literature

Other research^29^ broadly concurs with this study highlighting that VC/TC access does not facilitate an assessment of the patient’s physical state, for example being unable to determine whether they are out of breath, overweight, or able to assess their gait, as a patient walking into the consultation room allows. Further, there are lost opportunities including demonstrating exercises to reinforce the patient’s understanding of the therapy, which may result in non or incorrect compliance.^30^ Previous work has found that knowledge impacts whether PA is recommended or not.^31–33^

### Implications for research and practice

This study highlights that while GPs recognise the importance of regular PA for patients, many reported that lack of time, training and support impacted conversations around PA with patients. While previous research has identified the importance of knowledge, skills, and behavioural regulation for the ‘capability’ of behaviour change,^34^ acknowledging that improving GPs’ ability, knowledge, confidence, and ‘capacity’ to promote PA in routine practice for older adults is essential. This highlights the need for training for GPs to address health behaviour change for their patients and the need to combine PA with other health behaviours increasingly within an online context. There is the potential to further develop this at undergraduate training stages for GPs, especially increasing the understanding of the PA guidelines and benefits of PA, which would in turn help facilitate PA conversations during patient consultations in their practices whether in FTFC or VC/TCs.

It is evident that VC/TCs are beneficial to facilitate contact with many more patients than traditional FTFCs, and can potentially allow the GP more time to deal with specific patients if required. On the other hand, GPs suggested that VC/TCs may also have a negative impact, as with this type of consultation it is not possible to carry out a quick ’soft’ visual assessment, to determine any outward signs of health issues. Additionally, this may limit conversations around healthy lifestyle choices including the benefits of PA and the opportunity to signpost to local resources. Some GPs were worried that VC/TCs may have a detrimental impact on the doctor–patient relationship, and for some this extended to the relationship with the practice.^35^ Previous literature shows that practical interventions and conversations by GPs may improve engagement and participation in more active lifestyles.^18^ Importantly, with the exponential increase in use of VC/TC since the COVID-19 pandemic, evidence around access to and the use of VC/TC for marginalised and vulnerable populations is limited particularly for nursing homes, institutions, traveller populations, direct provision centres, and immigrant populations, thus highlighting the risk of widening health inequalities^28,36–38^ and compromising health equity and optimal health and wellbeing outcomes.

The full impact of remote consultations on patient outcomes remains unknown; however, this study found most GPs appear to favour this method. Further research is recommended to identify how this may impact vulnerable, disadvantaged, and marginalised groups. Evidence shows that VC/TCs in comparison with FTFCs, are more likely to be used by younger working people who are non-immigrants.^39^ It also shows that women consistently used more remote forms of consulting than men. In addition, internet-based consultations appear to be used more by younger, affluent, and educated groups. The evidence also suggested that TCs are used by the older population owing to issues of mobility or limited GP home visits.^39^

GPs agree that discussing PA is part of their job role and successful implementation and discussions around PA promotion in routine practice will result in considerable health benefits for the population, and for those older adults who may also benefit the most from increased PA levels.^40^ GPs also recognise the increasing demands on primary care services and proposals for VC/TCs to replace routine FTFCs in general practice. GPs recognise the importance of education and skill development to fully equip their knowledge of PA guidelines, to support their delivery, conversations, and discussions around PA with patients in a VC/TC setting, enabling information to be delivered both confidently and successfully.

## Data

The dataset generated and/or analysed during the current study is not publicly available.
